# Genomic and ecological evidence shed light on the recent demographic history of two related invasive insects

**DOI:** 10.1038/s41598-022-21548-y

**Published:** 2022-11-16

**Authors:** Daniel Poveda-Martínez, Nicolas A. Salinas, María Belén Aguirre, Andrés F. Sánchez-Restrepo, Stephen Hight, Hilda Díaz-Soltero, Guillermo Logarzo, Esteban Hasson

**Affiliations:** 1 Fundación Para El Estudio de Especies Invasivas (FuEDEI), Hurlingham, Argentina; 2 Facultad de Ciencias Exactas Y Naturales, Instituto de Ecología Genética Y Evolución de Buenos Aires (IEGEBA), Universidad de Buenos Aires, Buenos Aires, Argentina; 3 Consejo Nacional de Investigaciones Científicas y Técnicas (CONICET), Buenos Aires, Argentina; 4 Insect Behavior and Biocontrol Research Unit (IBBRU), USDA-ARS, Tallahassee, FL USA; 5 USDA, San Juan, PR USA

**Keywords:** Invasive species, Population genetics, Entomology

## Abstract

*Hypogeococcus pungens* is a species complex native to southern South America that is composed of at least five putative species, each one specialized in the use of different host plants. Two of these undescribed species were registered as invasive in Central and North America: Hyp-C is a cactophagous mealybug that became an important pest that threatens endemic cactus species in Puerto Rico, and Hyp-AP feeds on Amaranthaceae and Portulacaceae hosts, but does not produce severe damage to the host plants. We quantified genomic variation and investigated the demographic history of both invasive species by means of coalescent-based simulations using high throughput sequencing data. We also evaluated the incidence of host plant infestation produced by both species and used an ecological niche modeling approach to assess potential distribution under current and future climatic scenarios. Our genetic survey evinced the footprints of strong effective population size reduction and signals of genetic differentiation among populations within each species. Incidence of plant attacks varied between species and among populations within species, with some host plant species preferred over others. Ecological niche modeling suggested that under future climatic scenarios both species would expand their distribution ranges in Puerto Rico. These results provide valuable information for the design of efficient management and control strategies of the Puerto Rican cactus pest and shed light on the evolutionary pathways of biological invasions.

## Introduction

Invasive phytophagous insects are important drivers of ecological disturbances^1,2^. Their impacts directly affect native biodiversity by feeding on native plants and hybridizing with native species, or indirectly, through cascading effects, competing for food or space, carrying diseases, modifying parasitoid-host or predator–prey interactions, etc.^3^. In addition, invasive insects are responsible for causing extensive economic losses to agriculture^4,5^.

Improving the understanding of biological invasions with strategies combining genomics and ecological approaches have become feasible and popular, contributing useful information to the development of pest management strategies^6–9^. From an evolutionary perspective, the study of biological invasions provides the opportunity to elucidate the processes that permit the rapid evolution of invasive species which often occurs in the face of extremely low genetic variation and under novel ecological circumstances^10,11^. In this sense, genome-wide data provides information on several aspects of invasions such as source populations, colonization routes, demographic processes, including the time and intensity of founder effects and genetic bottlenecks^7,8,11^. Such events can shape the fate of introduced populations and have an impact on the observed spatial distribution of genetic variation. On the other hand, the study of patterns of host use in invasive herbivorous insects may also contribute to the understanding of the dynamics and history of biological invasions in new areas. Since invading insects may encounter novel hosts, host plant use in the new environment may differ from patterns in the native range^12^. Invading insects may expand their host range to other phylogenetically related host plants available in the new area, or exhibit some degree of specialization, depending on insect preferences and resource availability^13–16^. Likewise, approaches such as ecological niche modeling (ENM) allows the prediction of suitable conditions, potential spatial distributions and identification of potential areas of expansion of invasive species in a certain area or time, information that may contribute to pest management strategies^17–19^.

Here we focus on the mealybug *Hypogeococcus* sp. (Hemiptera: Pseudococcidae), a serious phytophagous pest affecting native cacti in Puerto Rico^20^. Seven out of the fourteen native cactus species reported in Puerto Rico^21^ are threatened by *Hypogeococcus* sp.^22^. The mealybug infestation promotes abnormal stem growth on infested cacti, including gall-like formations and flower deformation^22^, severely affecting the survival and reproduction of infested plants^20,23^. In the absence of control measures, the mealybug cactus pest will continue to decimate Puerto Rican cactus diversity and threaten other cactus rich ecosystems across the Caribbean islands, Central America and, potentially, North America^24,25^.

**Figure 1:**
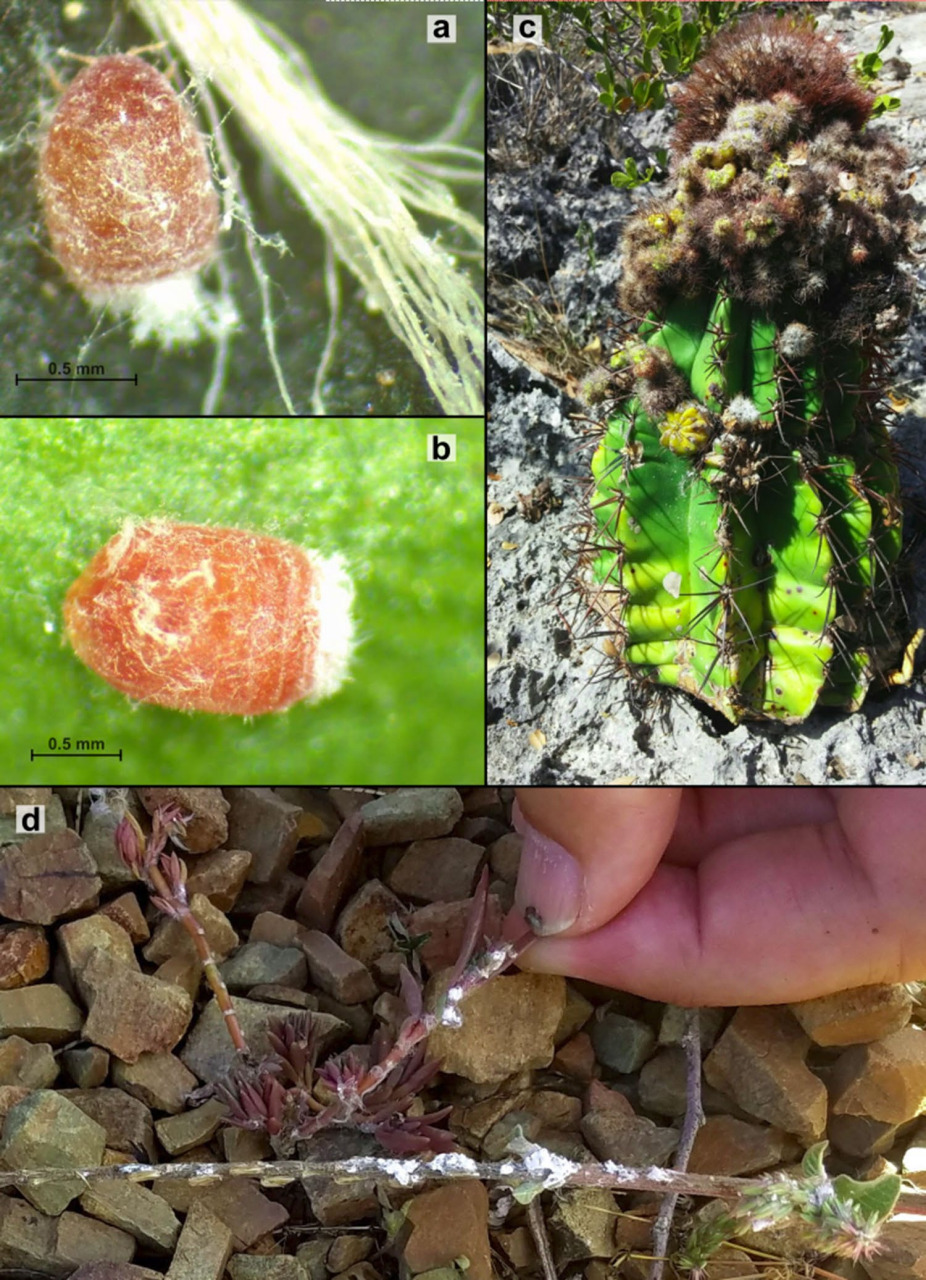
Adult females and host plant damage of both Hypogeococcus species invading Puerto Rico. Images showing adult females of Hyp-C (**a**) and Hyp-AP (**b**) as well as the typical damage of Hyp-C mealybugs on Cactaceae (**c**), and Hyp-AP on Amaranthaceae/Portulacaceae (**d**). Panel c shows advanced infestation by mealybugs causing deformation with shape of galls in a globular infested cactus. Plant held in the fingers of a researcher (**d**) shows the typical damage caused by Hyp-AP on Portulacaceae host plants, while the other plant on the lower panel d shows the damage on Amaranthaceae host plants.

The Puerto Rican cactus mealybug pest was formerly identified as *Hypogeococcus pungens* Granara de Willink^26^. However, it is now acknowledged that “*H. pungens*” is a species complex (i.e. a group of species very much alike in which the boundaries between putative species are often unclear), composed of at least five genetically differentiated putative species showing different patterns of host plant use^25^ and biology (MBA unpublished data). Recent genetic surveys based on single-locus nuclear and mitochondrial markers^27^, as well as genome-wide SNPs^25^, revealed two independent invasion events into Puerto Rico by two members of the *H. pungens* complex: a cactus feeding mealybug (Hyp-C) and mealybugs feeding on Amaranthaceae and Portulacaceae (Hyp-AP) (Fig. 1), both derived from putative source populations in Brazil^25^. Moreover, taxonomical studies are underway to formally describe both presumptive species, a necessary task for the adoption of appropriate pest management strategies.

How and when the invasions of Hyp-C and Hyp-AP occurred in the Caribbean and North America are still open questions. The presence of mealybugs feeding on cacti in Puerto Rico was first reported in 2005^26^. Historical records of invasive mealybugs attacking cacti in California date back to 1951, that were assigned to *Hypogeococcus spinosus* Ferris^28^; a species identified before the description of *H. pungens.* Another South American mealybug feeding on cacti identified as *Hypogeococcus festerianus* Lizer y Trelles, was reported in 1984 in Florida, United States of America (U.S.)^29^. However, the Florida invasive was later determined to be a misidentification of *H. pungens*^30^. Mealybugs identified as *H. pungens* feeding on Amaranthaceae and Portulacaceae were historically recorded on *Alternanthera ficoidea* (L.) Sm. at nurseries in Hialeah, Florida (U.S.) since 1996^31^. Later, this species was also reported for the first time in San Juan, Puerto Rico on the ornamental *Portulaca oleracea* L. in 2000^26^.

In this study, we used high-throughput sequence data to shed light on the history of the introduction of both Hyp-C and Hyp-AP putative species, in Puerto Rico. Although the latter does not hold a pest status in Puerto Rico, it was included in the study because it is a potential host of the South American parasitic wasps *Anagyrus cachamai* Triapitsyn, Logarzo & Aguirre and *Anagyrus lapachosus* Triapitsyn, Aguirre & Logarzo (Hymenoptera: Encyrtidae) selected as biological control agents against Hyp-C^32,33^. We first conducted population genetic analyses, quantifying genomic diversity and population structure in both Hyp-C and Hyp-AP. We then modeled the demographic histories of both putative species using coalescent-based simulations to investigate the time and intensity of presumptive founder events associated with the invasions. Third, we evaluated the incidence of host plant infestation and assessed whether the percentage of attack varied within and between mealybug species throughout the islands. Finally, we used an ecological niche modeling approach to investigate the potential distribution area of each species at the present time and under scenarios of future climate change.

## Results

### Genomic data

**Figure 2:**
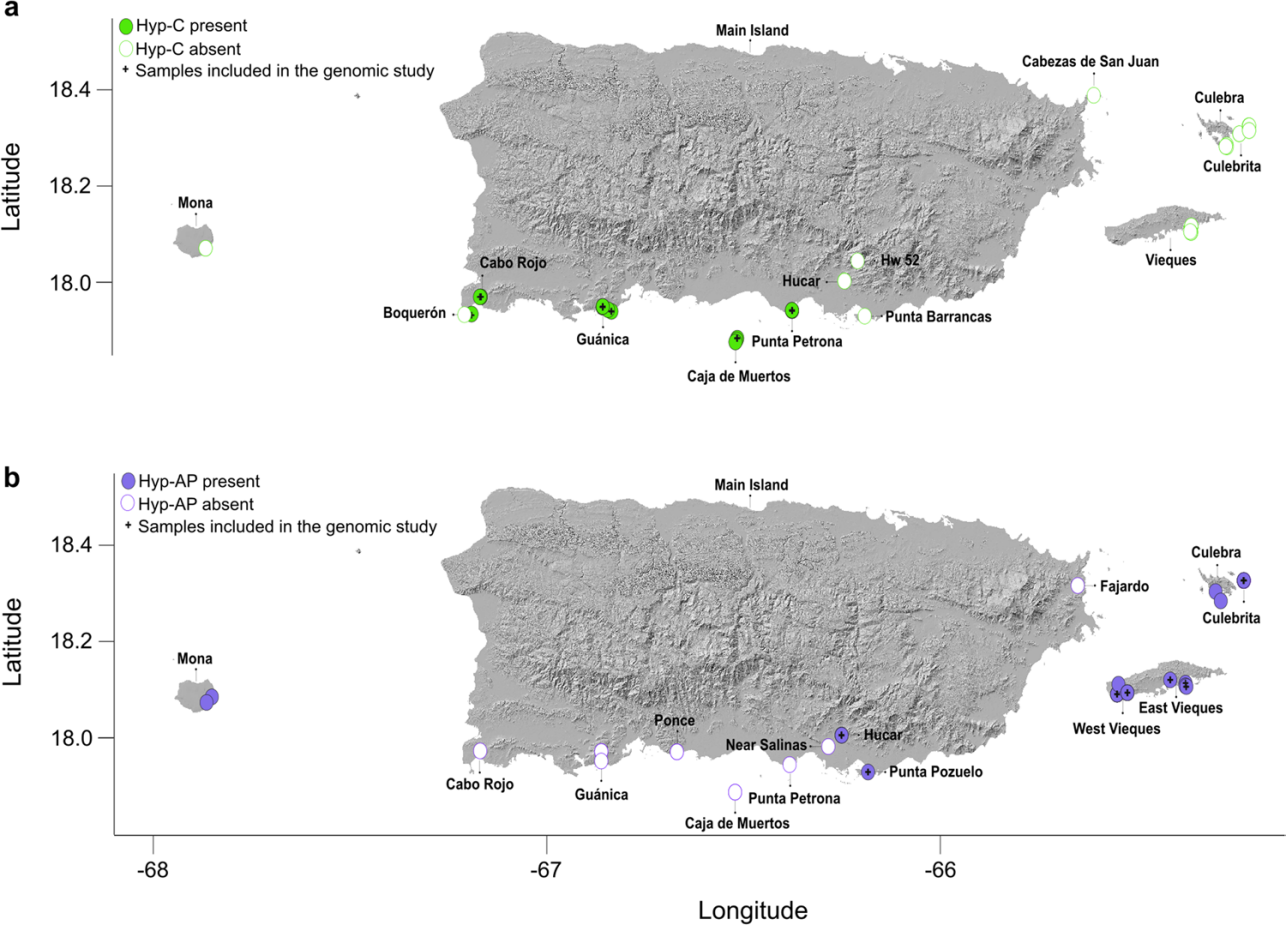
Geographic location where *Hypogeococcus* spp. mealybugs populations were sampled. Sites on Puerto Rican main island and its nearby outer islands (Vieques, Culebra, Culebrita, Caja de Muertos, and Mona) surveyed for presence of both *Hypogeococcus* species Hyp-C (**a**) and Hyp-AP (**b**).

An average number of 1.6 and 1.8 million single-end reads per individual was retained after quality control for 38 individuals of Hyp-C and 33 of Hyp-AP, respectively. Insects were sampled across the main island of Puerto Rico and its adjacent small islands (Fig. 2; Supplementary Table S1). Reference based assembly and variant calling executed in ipyrad v.0.9.71^34^ yielded 39,266 SNPs for Hyp-C and 61,270 SNPs for Hyp-AP. One individual of Hyp-C and two of Hyp-AP with large numbers of missing data (> 45% missing genotypes) were removed from the datasets in further analyses. Datasets without filtering rare variants were used for demographic analyses resulting in a matrix of 4,589 SNPs and 8,576 SNPs for Hyp-C and Hyp-AP, respectively, whereas filtered dataset used for population structure and diversity resulted in a matrix of 1,524 biallelic unlinked SNPs for Hyp-C and 1,284 for Hyp-AP with 32X and 33X average mean depth per individual, respectively. The numbers of loci removed in each filtering step are detailed in Supplementary Table S2 while final datasets used in all analyses, as well as R code and other relevant data, are available in Figshare repository^35^.

### Population diversity and differentiation

Patterns of genetic diversity were characterized on the basis of genome-wide SNP data. Overall, observed heterozygosity (H_O_) was higher than expected heterozygosity (H_E_) in all sampling sites (Table 1). Both observed and expected heterozygosity were greater in Hyp-C than in Hyp-AP, and analyses of allele richness (A_R_) and private alleles (P_A_) revealed that the highest values of A_R_ and P_A_ were detected in Guánica for Hyp-C and in Culebrita island for Hyp-AP. Tajima’s D test yielded negative values for all sampling sites in both species (Table 1). Estimates of genetic diversity were similar after normalizing to N = 5 for Hyp-C and N = 4 for Hyp-AP for all sampling sites (Supplementary Table S3).

**Table 1:** Summary statistics of genetic diversity for both *Hypogeococcus* species invading Puerto Rico (Hyp-C and Hyp-AP).

Population	N	H_E_	H_O_	A_R_	F_IS_ (CI95)	Tajima’s D (± SE)*	PA
**Hyp-C**							
Cabo Rojo	11	0.2	0.221	1210	− 0.097(− 0.419 − 0.097)	− 0.518 ± 0.021	39
Caja de Muertos	5	0.122	0.154	1137	− 0.224 (− 0.666 to − 0.077)	− 0.530 ± 0.030	12
Guánica	11	0.218	0.254	1229	− 0.131 (− 0.447–0.074)	− 0.458 ± 0.022	57
Punta Petrona	10	0.168	0.18	1177	− 0.081 (− 0.387–0.077)	− 0.689 ± 0.019	44
**Hyp-AP**							
Hucar	8	0.129	0.148	1139	− 0.117 ( − 0.409 to − 0.033)	− 1.033 ± 0.008	59
Vieques (W)	9	0.126	0.145	1134	− 0.122 (− 0.403 to − 0.014)	− 0.914 ± 0.015	40
Vieques (E)	4	0.11	0.132	1128	− 0.190 (-0.739 to − 0.094)	− 0.662 ± 0.026	16
Culebrita	10	0.161	0.178	1171	− 0.086 (− 0.359 to 0.030)	− 1.020 ± 0.009	118

N: sample size; H_E_: expected heterozygosity; H_O_: observed heterozygosity; A_R_: allelic richness; F_IS_: inbreeding coefficient, P_A_: private alleles.

**Figure 3:**
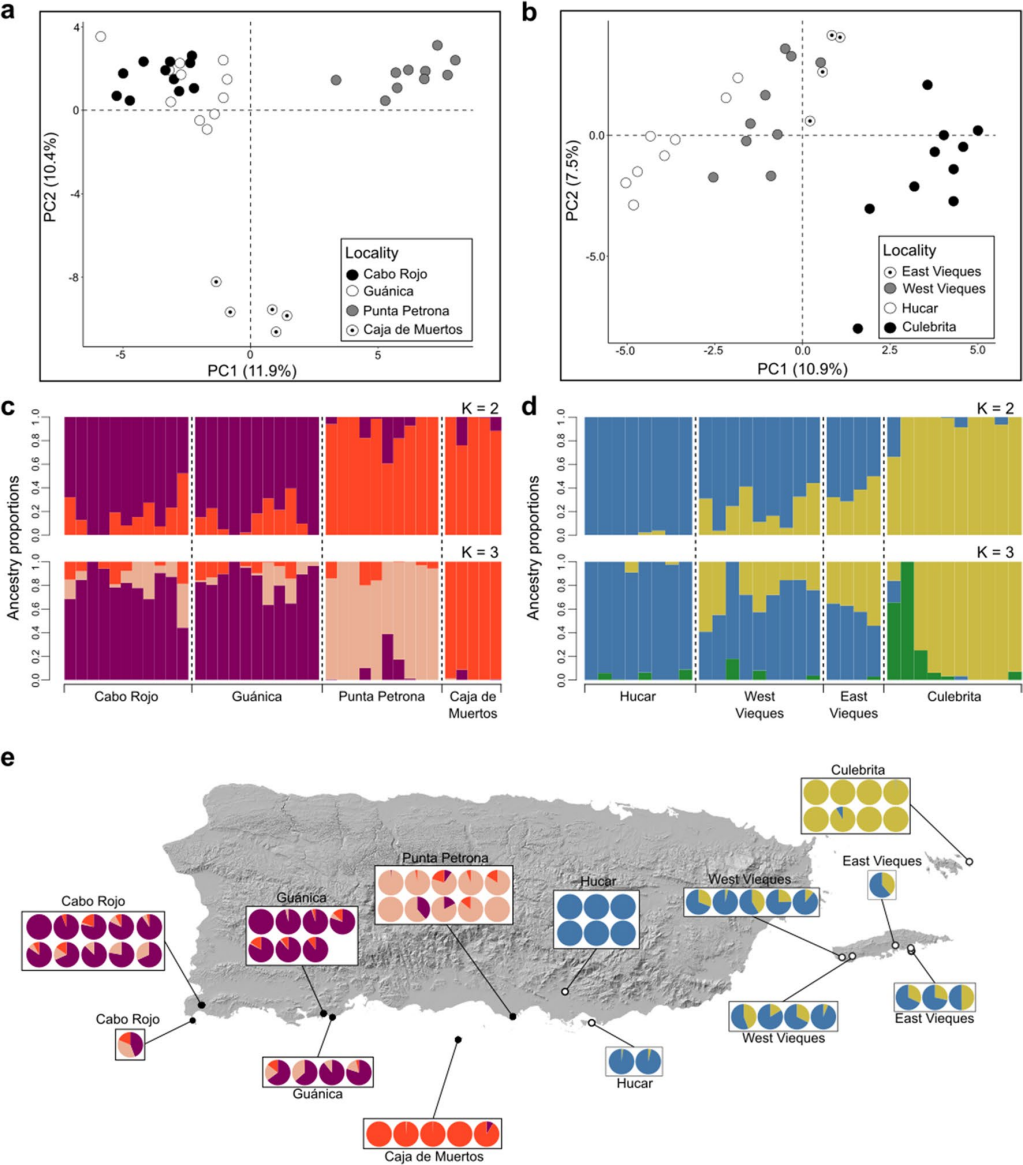
Population genetic structure of both *Hypogeococcus* species invading Puerto Rico. Visual representation of population genetic structure and individual ancestry coefficient of Hyp-C and Hyp-AP, using PCA (**a, b**) and sNMF (**c–e**), respectively. PCA shows the grouping of three subpopulations belonging to the Puerto Rico mealybug cactus pest (**a**), and two main subpopulations representing Hyp-AP (**b**). Vertical bars in panels c and d represent individuals along with membership probability of each individual to the corresponding subpopulations in Hyp-C and Hyp-AP, respectively. Panel e shows ancestry coefficients for each individual of both putative species plotted on a map of Puerto Rico.

Principal component analysis (PCA) revealed that the first two axes (PCs) accounted for 22.3% and 18.4% of observed genomic variation in Hyp-C and Hyp-AP, respectively (Fig. 3). Hyp-C mealybugs collected in Punta Petrona, and Caja de Muertos island formed two distinct groups whereas specimens from Cabo Rojo and Guánica, formed a third cluster (Fig. 3a). Hyp-AP mealybugs collected on Culebrita island were differentiated from the other sampling sites, while mealybugs from Hucar-Punta Pozuelo (Hucar from hereafter), the only population on the main island, appeared close to both Vieques populations (Fig. 3b). After removing population-specific SNPs (private alleles) from the dataset, the new PCAs remained qualitatively the same for both species (Supplementary Fig. S1).

Admixture analyses using sNMF^36^ also revealed signals of geographic structure in both species. The optimal K value according to the cross-entropy criterion was K = 2 for Hyp-C, with Punta Petrona and Caja de Muertos island separated from Guánica and Cabo Rojo. Considering K = 3, the first two sampling sites appeared as two distinct groups, while Guánica and Cabo Rojo remained together, forming a unique cluster with a certain degree of admixture (Fig. 3c–e). For Hyp-AP, K = 1 was the optimal value. With K = 2, Hucar and Culebrita, the most geographically distant locations, appeared well differentiated, meanwhile, Vieques (East and West), located halfway between Hucar and Culebrita island, exhibited a mixture of genotypes of both populations (Fig. 3d–e).

Pairwise F_ST_ between sampling localities showed varying degrees of differentiation in both species. F_ST_ ranged from 0.017 to 0.230 in Hyp-C, and from 0.026 to 0.113 in Hyp-AP (Table 2). In the former, the highest F_ST_ values were found between Caja de Muertos and the rest of the collecting sites, and the lowest differentiation occurred between Guánica and Cabo Rojo. In Hyp-AP, the highest F_ST_ was found between Hucar and Culebrita island, while East Vieques and West Vieques island were less differentiated.

**Table 2:** Pairwise F_ST_ values estimated between collection sites of both *Hypogeococcus* species (Hyp-C and Hyp-AP) using Wright and Cockerham method.

Hyp-C	Caja de muertos	Cabo rojo	Guánica
Cabo Rojo	0.181		
Guánica	0.149	0.017	
Punta Petrona	0.23	0.134	0.103

Hyp-AP	Vieques (W)	Vieques (E)	Culebrita
Vieques (E)	0.026		
Culebrita	0.067	0.05	
Hucar	0.044	0.074	0.113

### Demographic history of both invasions

**Figure 4:**
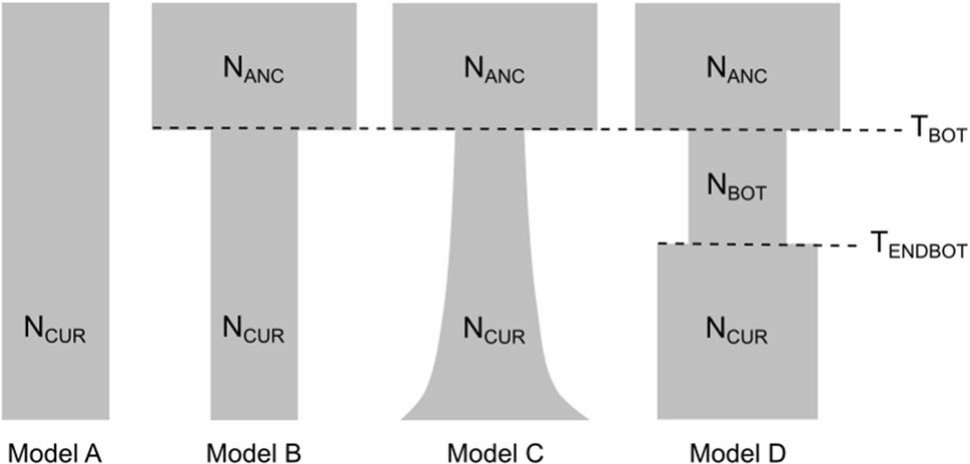
Schematic representation of demographic models. Estimated parameters of demographic models using Fastsimcoal2 included timing of bottleneck (T_BOT_ and T_ENDBOT_), historical effective population sizes (N_ANC_, N_BOT_) and exponential growth rate (G_R_). Contemporary effective population size (N_CUR_) was fixed in the models.

Alternative demographic models were investigated by means of coalescent-based simulations using Fastsimcoal2^37^. In both putative species, we simulated four scenarios using a single population demographic model (see Methods section for details on the models used; Fig. 4). Two models (B and C) were selected out of the four models proposed to investigate the demographic history of both invasive species, on the basis of AIC. We could not select a unique model since the difference in AIC values between models was lower than three units (Supplementary Table S4). In Hyp-C, model B was selected in 74% of the iterations, and model C in 26%. The same pattern was observed in Hyp-AP: model B was the most frequently selected in 86% of the iterations, and model C in the remaining 14%. Parameter estimates under both supported models (B and C) indicated severe population size reductions of over 80% and 95% of the ancestral effective population size during the invasions of Hyp-C and Hyp-AP, respectively (Table 3). In model C, the growth rate parameter (*r*) was negative for both species, *r* = −1.72 × 10^–6^ for Hyp-C, and *r* = −1.55 × 10^–5^ for Hyp-AP. Since growth rates are measured backwards in time, such negative growth rates are indicative of population expansions^41^. Considering an average of 5.5 generations per year as reported in other *Hypogeococcus* species feeding on Cactaceae and Amaranthaceae-Portulacaceae in Argentina (MBA unpublished data), the results of Fastsimcoal2 simulations suggest that the introduction of Hyp-C probably occurred ~ 21 years ago [95% Confidence interval, (95% CI) = 12–43], and ~ 39 years ago [95% CI = 30–49] for Hyp-AP (Table 3).

**Table 3:** Point estimates of demographic history of two invasive *Hypogeococcus* species and 95% confidence intervals of parameters inferred from coalescent simulations using Fastsimcoal2. Point estimates included parameters for the two models fitting our data, such as timing of effective population size reduction given in number of generations (T_BOT_), historical effective population size (N_ANC_), and growth rate (*r*). Contemporary effective population size was fixed in the models (Hyp-C, N_CUR_ = 206,750; Hyp-AP, N_CUR_ = 48,600).

Parameter	Point estimate	95% CI
**Model B**		
**Hyp-C**		
N_ANC_	1,044,983	1,044,700–1,067,592
T_BOT_	114	61–239
**Hyp-AP**		
N_ANC_	1,044,710	1,044,547–1,075,916
T_BOT_	217	167–271
**Model C**		
**Hyp-C**		
N_ANC_	1,045,038	1,044,158–1,087,345
T_BOT_	115	73–308
*r*	− 1.72 × 10^–6^	− 7.82 × 10^–6^ to − 1.4 × 10^–5^
**Hyp-AP**		
N_ANC_	1,044,784	1,044,639–1,087,032
T_BOT_	217	168–255
*r*	− 1.5 × 10^–5^	− 9.09 × 10^–5^ to − 1.44 × 10^–5^

### Assessment of host plant infestation

A total of 4,563 plants (2058 Cactaceae, 1544 Portulacaceae, and 961 Amaranthaceae) were inspected in search of mealybugs. Hyp-C was found in Caja de Muertos island and at three sites in the main island, while Hyp-AP was found in the outer islands (Culebra, Culebrita, Vieques, Mona), and in two sites in the main Puerto Rico island (Fig. 2). Hyp-C was detected on 268 out of 445 cactus plants examined (60%) while Hyp-AP on 451 out of 1,883 Amaranthaceae-Portulacaceae inspected (24%), a difference that was significant (quasibinomial GLM; *F* = 11.223, *df* = 1, *P* < 0.01).

Host plant use in Hyp-C varied according to geographic area (*X*^2^ = 61.9, *df* = 3, *P* < 0.01). Guánica, Cabo Rojo, and Punta Petrona were the sites that showed the highest proportion of infested plants. In each locality, we computed the proportion of available plants and estimated the 95% confidence intervals of the proportions of plants used. The analysis of these results showed that in Guánica [proportion of plants available (PPA) = 0.299; confidence interval of proportion of plant use (CI) = 0.221–0.376] and Cabo Rojo (PPA = 0.099, CI = 0.074–0.187) plants were attacked according to their availability, while plants were attacked more than expected by chance in Punta Petrona (PPA = 0.378, CI = 0.450–0.618). Caja de Muertos island had the lowest number of infested plants, and plants were attacked less than predicted based on their availability (PPA = 0.225, CI = 0.005–0.069). Hyp-C showed a marked preference for *Pilosocereus royenii* (L.) Byles & Rowley (PPA = 0.470, CI = 0.657–0.806), followed by *Melocactus intortus* (Mill.) Urb. (PPA = 0.200, CI = 0.083–0.201) and *Hylocereus trigonus* (Haw.) Saff. (PPA = 0.094, CI = 0.028–0.114). These three host plants were used proportionally to their abundance, while *Stenocereus fimbriatus* (Lam.) Lourteig (PPA = 0.236, CI = 0.017–0.095) tended to be avoided (*X*^2^ = 81.975, *df* = 3, *P* < 0.01) (Supplementary Table S5).

Regarding numbers of host plants attacked by Hyp-AP, differences were also found among sites (*X*^2^ = 69.810, *df* = 6, *P* < 0.01). Culebrita and Hucar were the sites with the highest proportions of infested plants, followed by Vieques (East and West) and Culebra islands, while Mona island and Punta Pozuelo showed the lowest numbers of plants attacked. In both Culebrita (PPA = 0.115, CI = 0.127–0.233) and Hucar (PPA = 0.110, CI = 0.137–0.245), plants were more attacked than expected by chance, whereas in Culebra (PPA = 0.010, CI = −0.004–0.022), and Vieques East and West (PPA_E_ = 0.183, CI_E_ = 0.111–0.213; PPA_W_ = 0.384, CI_W_ = 0.291–0.423) plants were used in proportion to their availability. Mona (PPA = 0.164, CI = 0.058–0.141) and Punta Pozuelo (PPA = 0.035, CI = −0.004–0.009) were different in that plants were less attacked than expected by chance. The analysis of host plant infestation data showed that Hyp-AP had a marked preference for plants of the genus *Portulaca*: *Portulaca cf pilosa* L. (PPA = 0.073, CI = 0.074–0.166), *Portulaca rubricaulis* Kunth (PPA = 0.166, CI = 0.119–0.227) and *Portulaca teretifolia* Kunth (PPA = 0.263, CI = 0.313–0.450). Attacks to *Alternanthera crucis* Bold (PPA = 0.040, CI = 0.026–0.096) occurred according to availability, whereas *Achyranthes aspera* L. var. *aspera* (PPA = 0.318, CI = 0.165–0.283), *Gomphrena serrata* L. (PPA = 0.019, CI = −0.004–0.009), *Portulaca caulerpoides* Britton & Wilson (PPA = 0.106, CI = 0.001–0.043), *Portulaca oleracea* L. (PPA = 0.050, CI = −0.001–0.036), and *Portulaca* sp. (PPA = 0.016, CI = 0.000–0.000) tended to be avoided (*X*^2^ = 120.656, *df* = 8, *P* < 0.01) (Supplementary Table S5).

### Ecological niche modeling

In total, 16 occurrence records were used for Hyp-C and 18 for Hyp-AP. In model calibration, 341 models were evaluated per species using four variables: bio08; mean temperature of the wettest annual quarter, bio13; precipitation of wettest month, aridIndx; Thornthwaite aridity index, and annPET; annual potential evapotranspiration (selection process and results are detailed in Supplementary Methods S1).

**Figure 5:**
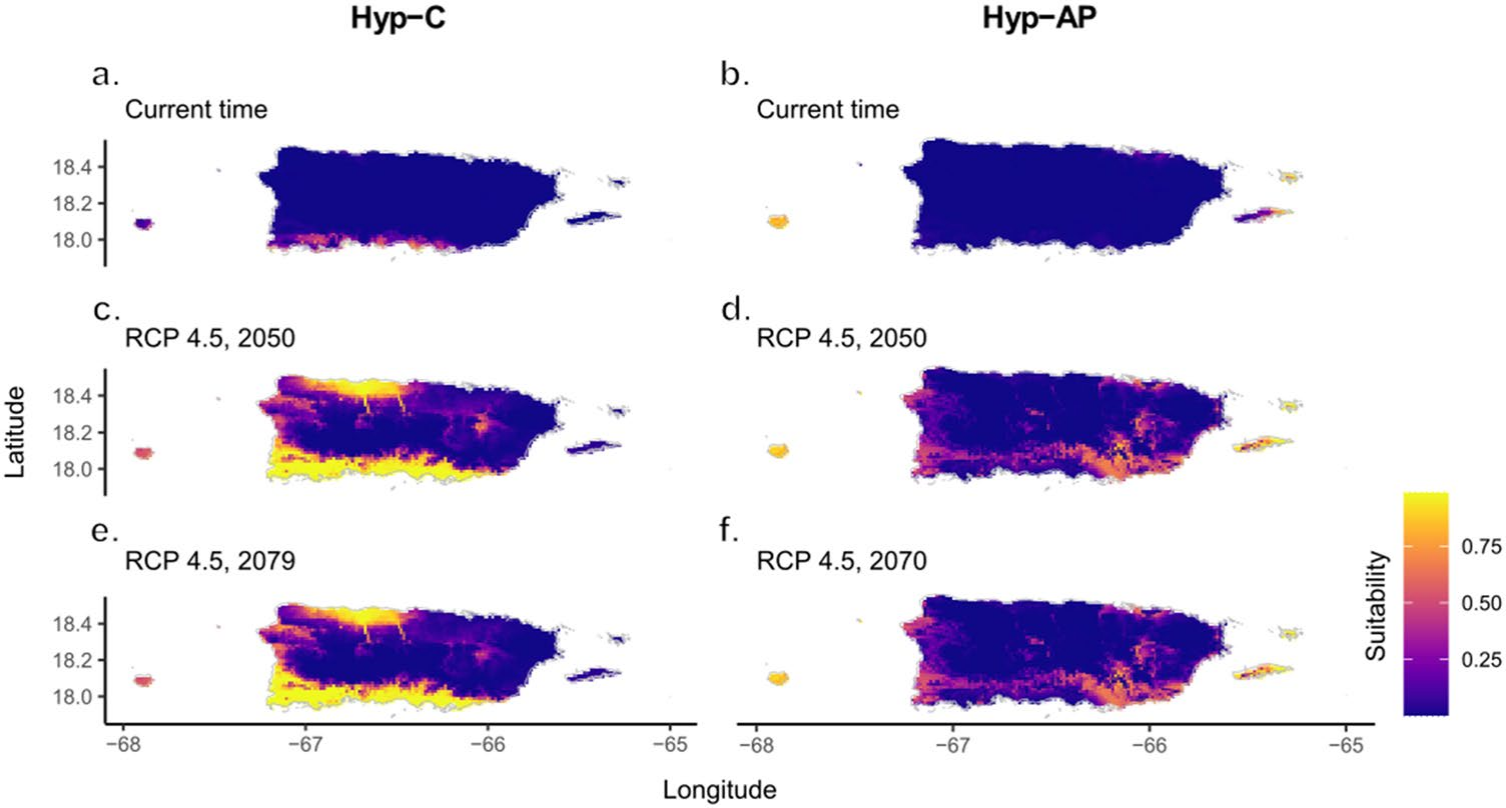
Ecological niche models of both *Hypogeococcus* species invading Puerto Rico. Maps indicate the suitability values of the best model of Hyp-C (**a**) and Hyp-AP (**b**) for the current time, future intermediate scenario (RCP 4.5) for the years 2050 (average for 2041–2060) (**c, d**) and 2070 (average for 2061–2080) (**e, f**).

Overall, all models were statistically significant when compared to a null random prediction model. The number of significant models that met the 5% omission criterion, the AICc criteria and the statistically significant models for both species are presented in Supplementary Table S6. Three models were selected for Hyp-C, all with area under the curve (AUC) > 0.99. The variables that contributed most to Hyp-C models were mean temperature of the wettest quarter (bio08, ~ 32%) and Thornthwaite aridity index (aridIndx; ~ 29%). The regions with the highest suitability values currently appear concentrated on the south coast of the main island of Puerto Rico (Fig. 5a). Future projections indicated new potential areas from the south coast towards the interior of the island, and a large area with high suitability values in the north (Fig. 5c and e). Niche modeling analyses also indicated suitability values for Mona Island, even though Hyp-C was not found in this island. For Hyp-AP, three models were also selected, all with AUC values greater than 0.97. The variables that most contributed to the Hyp-AP models were precipitation of the wettest month (bio13; ~ 58%) and Thornthwaite aridity index (aridIndx; ~ 16%). Currently, the regions with the highest suitability values were concentrated in the islands of Vieques, Culebra and Mona (Fig. 5b). Future projections indicated new potential areas from the southeast to the southwest coasts of the main island of Puerto Rico (Fig. 5d and f). The overlap identity test using "Schoener's D" and "Hellinger's I" showed that the niche models of Hyp-C and Hyp-AP do not overlap (Supplementary Figure S2).

## Discussion

Our survey of genomic variation combined with coalescent demographic modeling of both invasive mealybugs in Puerto Rico revealed that these related and still undescribed species (Hyp-C and Hyp-AP) went through severe genetic bottlenecks upon invasion. Genomic data also suggested that after spreading across the Puerto Rican Archipelago, genetic differentiation among populations evolved rapidly within each presumptive species. In addition, our survey of host plant use revealed that the proportions of infested plants varied not only between species, but also within species across their respective ranges. Our results indicated that both species chose to attack certain host plants rather than at random, suggesting some degree of preference. Ecological niche models indicated that Hyp-C and Hyp-AP not only differed in their current geographic distributions, but also that their current climatic niches do not overlap, indicating that their ecological requirements are different. Niche modeling analyses predicted that under future scenarios of climate change, Hyp-C and Hyp-AP would spread to new areas, posing an even greater threat for cactus conservation. Overall, these findings not only reveal the current genetic state of these invasions but are also a warning of the potential of both species spread across the region and neighboring areas.

The most supported demographic models (Models B and C) based on coalescent simulations suggested that after invasion both species experienced strong genetic bottlenecks, causing reductions of over 80% and 95% of the ancestral effective population size in Hyp-C and Hyp-AP, respectively. The higher observed heterozygosity than expected from the observed allele number at mutation-drift equilibrium could be considered as indicative of recent reduction in effective population size in concordance with demographic modeling. Overall, these results are in line with previous studies showing that nuclear and mitochondrial genetic diversity of Puerto Rican populations of Hyp-C and Hyp-AP are considerably lower than in putative source populations from Brazil^25^. However, models B and C propose different demographic scenarios after the genetic bottlenecks. While Model B supported a constant effective population size over time, the negative growth rate calculated in Model C suggests that both invasive mealybugs likely experienced exponential demographic growth after invasion. The fact that it was not possible to select a unique model based on AIC indicated that our study systems shared characteristics with both selected models. In this vein, the negative values of Tajima’s test statistic found in all Hyp-C and Hyp-AP sampling sites (Table 1) pointed to an excess of low frequency variants, which are the hallmark of population expansions^38^, as has been reported in other invasive species^39^. Thus, the combined evidence suggests a clear population size reduction as well as signals of ongoing population size growth in both species invading Puerto Rico.

Population establishment starting from just a handful of individuals has also been observed in other species of *Hypogeococcus*. This is the case of an undescribed mealybug of the *Hypogeococcus pungens* species complex that was extensively used as a biological control agent to mitigate the effects of cacti invasions in Australia^40,41^ and South Africa in the 1970s^42–44^. This mealybug was released in Australia in 1975, and by 1978 it had caused significant damage to cacti^45^. This successful biological control program demonstrates the high capacity of this mealybug to grow under novel ecological circumstances with a relatively low effort (a few ad hoc releases)^45–47^. Recently, the demographic histories of other biological invasions have also been studied using coalescent demographic modeling^11,47,48^. For instance, the invasion of the Western Mediterranean by the North American boatman *Trichocorixa verticalis verticalis* Fieber (Hemiptera: Corixidae) involved a severe effective population size reduction, followed by an almost instantaneous demographic expansion shortly after introduction, demonstrating the great capacity of this bug to recover^11^.

Coalescent-based modeling suggested that bottlenecks took place approximately ~ 114 (~ 21 years) and ~ 217 (~ 39 years) generations before our surveys of Hyp-C and Hyp-AP, respectively. For the former, bottleneck estimation dates to ~ 1996, nine years before the first report of this mealybug attacking cacti in Puerto Rico^26^, suggesting that the bottleneck occurred upon arrival to the island. In turn, demographic modeling dates Hyp-AP bottleneck back to ~ 1978, long before it was first found in Puerto Rico in 2000^26^. A possible explanation of these results is that this invasive mealybug went undetected for a longer time period than Hyp-C, because the damage that Hyp-AP produces on its specific hosts (Amaranthaceae and Portulacaceae) is more difficult to detect since the insect often settles in the roots or in the axils of the plants (Fig. 1d), or that it followed a more complex colonization route since leaving its native range until its arrival to Puerto Rico (e.g., passing through successive bottlenecks). The first detection of Hyp-C in Puerto Rico was reported in Guánica, and it was soon found along most of the south coast of the main island^26^. By 2014, the pest reached the small offshore island of Caja de Muertos (Chardon Aviles, personal communication). These historical records are in line with surveys of incidence of attacks conducted by Segarra-Carmona et al.^26^ between 2007 and 2009 which showed that the extent of damage produced by the cactus pest varied across locations, and that the highest incidence was observed in Guánica, the locality where the first infested cacti were reported. Our field surveys conducted in 2016 and 2018 also revealed high incidence of infestation in Guánica, Cabo Rojo and Punta Petrona, contrasting with Caja de Muertos Island, the latest area where Hyp-C was recorded, where the infestation was lower.

During the expansion stage of a biological invasion, varying levels of genetic diversity across locations are common. During spread and colonization, new populations may suffer successive founder effects and bottlenecks leading to further reductions in genetic diversity^8^. Areas exhibiting the highest genetic diversity corresponded to those where Hyp-C was first detected and exhibited the highest incidence of infestation, while the lowest records of infestation and genetic diversity were observed in Caja de Muertos. These findings support a scenario of a single introduction of Hyp-C into Puerto Rico (probably starting in the area of Guánica). Although alternative scenarios involving independent introductions cannot be ruled out, the finding of a single mitochondrial haplotype in Hyp-C^27^ along with the observed patterns of genetic diversity and plant infestation, gave support to the hypothesis of a single introduction in Puerto Rico**.**

Our genomic data also revealed early signals of intraspecific differentiation among Hyp-C populations. Mealybugs from Guánica and Cabo Rojo, located approximately 30 km apart from each other, appeared as a single panmictic unit. Lack of differentiation between these two sampling sites may reflect intense gene flow, probably promoted by environmental suitability and high cactus availability in the southern portion of the main island serving as a biological corridor or by human activity. In contrast, mealybugs from Punta Petrona, located further away on the coast of the main island, and from the single sampled population outside the main island (Caja de Muertos) formed two separate clusters in both sNMF and PCA analyses. Caja de Muertos was genetically differentiated despite that this small island represents the most recently colonized site, and the relative geographic proximity to Punta Petrona (approx. 13 km) (Fig. 2). These results suggest that the sea is a barrier to dispersion between Caja de Muertos and the main island. Although there is a certain degree of overlap in the distributions of host cacti across sampling sites, host plant preferences and divergent selection may be an additional ecological factor promoting genetic differentiation as shown in the native range of the *H. pungens* species complex^25^. Host plant use and geographic isolation seem to be the main forces promoting intraspecific divergence in herbivorous insects^49–51^. Rapid genetic differentiation among invasive insect populations has recently been reported in the cotton mealybug *Phenacoccus solenopsis* Tinsley (Hemiptera: Pseudococcidae). Demographic analyses based on SNPs data have shown that invasive populations of *P. solenopsis* derive from a single source population and exhibit limited gene flow and strong differentiation that evolved after introduction, suggesting adaptation to diverse climate regimes^10^. Likewise, rapid differentiation was also observed in the fall webworm, *Hyphantria cunea* (Drury) (Lepidoptera: Arctiidae), in China, where it was attributed to geographical barriers limiting gene flow^52^.

In contrast, Hyp-AP showed less genomic variation and a weaker population structure than Hyp-C. This may be related to the stronger population size reduction observed in Hyp-AP compared to Hyp-C. Demographic Model C evidenced a slower population growth for Hyp-AP following the bottleneck. Differences in the incidence of plant infestation (60% in Hyp-C and 24% in Hyp-AP) may account, at least in part, for the distinct demographic histories of both species. In this context, novel interactions between an herbivore and potential new hosts may affect the dynamics of the invasion^53^ and could propitiate demographic changes, genetic differentiation, and, eventually, the evolution of reproductive isolation associated with habitat preference and host plant use. However, additional studies including whole genome sequencing and annotation are necessary to disentangle the factors underlying rapid genetic differentiation in both mealybug species.

Although niche modeling analyses indicated that current climatic niches do not overlap between both invasive species, the models also depict a very plausible scenario in which both Hyp-C and Hyp-AP would spread to new areas and become sympatric under ongoing trends of climate change. Such range overlap may negatively affect management strategies since sympatry could facilitate hybridization, a frequent event documented in closely related species^51,54,55^. This hypothetical scenario would bring about a potential source of new genetic variation prompting adaptive changes in the recipient species^56^, triggering adaptive radiation in the cactus pest. However, a word of caution is needed before arriving at definitive conclusions due to limitations of climatic niche models which were built using a low number of occurence records. Although this is considered a limitation on niche modeling, models were consistent with the expected distribution range of both species in Puerto Rico islands. Climatic models also indicated that ecological requirements of both species are different, despite both being distributed along dry forests of Puerto Rico, an area characterized by scarce rainfall, suggesting that variables related to humidity are limiting the ranges of the host plants and both mealybug species.

In 2010, a biological control program was implemented in Puerto Rico to reduce the effect of the pests on cactus diversity. This management strategy is based on the release of biological control agents (i.e., parasitoids) that may affect mortality and reproductive rates of the target pest. Many examples of biological control demonstrated the success of parasitoids in reducing the harm produced by invasive insects^57–59^. Currently, there is an ongoing search for additional species-specific parasitoids to control the cactus pest, along with the development of mathematical models to assess the number of species that should be released, the order of release, and their potential impact on host suppression^32,33^.

In conclusion, our results indicate not only a clear population size reduction upon invasion in both invasive species, but also signals of ongoing population size growth and dispersal, accompanied by early intra-specific differentiation. Hyp-C showed a higher incidence of infestation than Hyp-AP. Moreover, both mealybug species preferred some host plant species over others. Because Hyp-C kills its host cacti, it is urgent to protect Puerto Rican cacti species potentially affected by the pest and threatened with extinction because of its impact. Thus, our study provides valuable information that should be taken into account in the design of efficient management and control strategies for the Puerto Rican cactus pest.

## Methods

### Study area

Surveys were carried out in Puerto Rico, including nearby outer islands (Vieques, Culebra, Culebrita, Caja de Muertos, and Mona), in February 2016 and April 2018 focusing on the host plant distributions of Hyp-C (Cactaceae) and Hyp-AP (Amaranthaceae and Portulacaceae). Overall, the Puerto Rican Archipelago is composed of different ecosystems including subtropical dry and rain forests from low-land to lower montane forests. Most of them occur in different stages of succession including those fragmented by human activity or others recovering from hurricanes^60^. Hyp-C and Hyp-AP mealybug populations were mostly distributed along the subtropical dry forest characterized by scarce rainfall (mean annual rainfall ranges from a minimum of about 600 mm to a maximum of 1100 mm)^60^ located along the South of Puerto Rico main island, Vieques as well as Mona and Culebra islands. While Hyp-C was mostly distributed in natural or protected areas, Hyp-AP was also found in disturbed areas such as parking lots, sidewalks, and on the side of roads.

Sampled sites were selected based on observation of host plants while driving along highways, roads, and protected natural areas. Stops were made when potential host plants (Amaranthaceae, Portulacaceae, and Cactaceae) were observed. The minimum distance between stops was 2 km. In areas where we did not find host plants, we stopped every 10 km, and inspected the area for the presence of potential host plants (the island territory is 160 km long and 35 km wide). In each sampling site, two trained persons searched for the presence of Hyp-C/Hyp-AP for 60 min, recording the number of plants examined and the number of plants attacked by Hyp-C or Hyp-AP.

### Population sampling and genotyping

For the genetic survey, adult females of each species were collected in the field directly from the host plants, Cactaceae and Amaranthaceae/Portulacaceae for Hyp-C and Hyp-AP, respectively. Samples of Hyp-C were collected in Guánica, Punta Petrona, Caja de Muertos, and Cabo Rojo, and Hyp-AP in Culebrita, Vieques, Hucar and Punta Pozuelo. Adult females from each location were transferred to a vial containing 100% ethanol for DNA extraction and posterior NextRAD sequencing and draft genome assembly (see Poveda-Martínez et al.^25^ for further details on DNA extraction and sequencing procedures). High throughput sequencing data were obtained from mealybugs feeding on 38 cacti (Hyp-C) and 33 Amaranthaceae-Portulacaceae (Hyp-AP) (Fig. 2). Raw sequence data were deposited in the National Center for Biotechnology Information (NCBI) (BioProject accession number: PRJNA593002).

### Genomic data processing

Demultiplexed raw reads from NextRAD sequencing were first pruned to remove Nextera adaptors using Trimmomatic^61^. Read quality and length were assessed for each sample with FastQC^62^ and outputs compiled and summarized on MultiQC v1.10.1^63^. Based on these reports, reads were processed with ipyrad v.0.9.71, trimming the first 12 bases of each read, allowing a minimum and maximum read length of 50 bp and 145 bp, respectively, and a Phred quality score > 33. Filtered reads were mapped against *Hypogeococcus pungens'* draft genome (NCBI accession number: JAAOIU000000000). On subsequent ipyrad assembly steps, 20% of SNPs per locus were allowed, polymorphic sites occurring across a maximum of 25% of samples, and minimum number of samples per locus representing 25% of the total. The resulting Variant Call Format (VCF) files for Hyp-C and Hyp-AP were then filtered using VCFtools v.0.1.16^64^ based on the following criteria: maximum number of alleles (2), maximum missing data per site (< 25%) and read depth per site (min = 6X–max = 100X). Additionally, individuals with more than 45% missing data and monomorphic SNPs were excluded for further analysis. To prune SNPs in linkage disequilibrium we used PLINK v1.9^65^ with recommended parameters: window size (50), window shift (5) and VIF threshold (2). Lastly, SNPs under selection were identified using Bayescan v.2.1^66^ and excluded from the dataset. We generated two datasets that differed in an additional filtering step of rare alleles. Since rare variants are necessary to infer recent coalescent demographic events, we generated one dataset keeping all rare variants without filtering on minor allele frequency (MAF). Meanwhile, since model-based inference of population structure has been shown to be confounded when singletons are included in the alignments^67^, a second dataset was built using a MAF < 0.03 cutoff for genetic diversity and population structure analyses.

### Genetic diversity and population structure

Patterns of genetic diversity in both putative species for each sampling locality were characterized by means of expected heterozygosity (H_E_), observed heterozygosity (H_O_), allele richness (A_R_) and number of private alleles (P_A_). However, samples collected in Hucar and Punta Pozuelo were pooled due to low sample size. Both H_E_ and H_O_, as well as the inbreeding coefficient (F_IS_), were calculated with the R package *diveRsity*^68^ with 9,999 bootstrap replicates, while A_R_ and P_A_ were computed with *hierfstat*^69^ and *poppr*^70^ respectively. Tajima’s D test^71^, calculated using VCFtools in 10-Mbp windows, was employed to test for departures from expectations under neutral models.

Population genetic structure was investigated using principal component analysis (PCA) performed with R package *hierfstat*^69^ for each species, and approximated by the first two principal components. To determine whether P_A_ influenced population genetic structure analyses, previously identified SNPs with population-specific alleles were excluded from the datasets and PCAs were repeated with these filtered datasets. The sparse non-negative matrix factorization approach (sNMF)^36^, as implemented in *LEA* R package^72^, was used to assign samples to genetic clusters through the estimation of individual admixture coefficients. Such coefficients were calculated for *K* values ranging from 1 to 10, with 100 repetitions for each value of *K* and 9,999 iterations. An optimal *K* value was assessed using the cross-entropy criterion based on the prediction of masked genotypes to evaluate the error of ancestry estimation. Cross-entropy was only used to find the *K* value with best predictive accuracy but not to determine an absolute *K*. Pairwise fixation indexes between sampling localities (pairwise F_ST_) were calculated using R package *StAMPP*^73^*,* according to the method proposed by Weir and Cockerham^74^, with 9,999 bootstrap replicates.

### Demographic history

A simulation-based approach was employed to investigate the occurrence and magnitude of presumptive bottlenecks experienced by each species upon invasion and posterior recovery through coalescent simulation using Fastsimcoal2^37^. Four alternative single-population demographic models were examined which allowed a further investigation of the demographic history of each invasive species (e.g.^11,48^). As a null model (model A), a scenario of constant effective population size was used over time. Other models simulated scenarios of population size reduction followed by constant effective population size over time (model B), population size reduction followed by exponential expansion (model C) and a full model of genetic bottleneck considering processes such as population size reduction, duration of the genetic bottleneck and immediate demographic expansion (model D) (Fig. 4). Demographic parameters, such as the timing of the bottleneck (T_BOT_, T_ENBOT_), ancestral effective population size (N_ANC_), population size during bottleneck (N_BOT_), as well as exponential growth rates (G_R_), were estimated from the models using the Site Frequency Spectrum (SFS) based on SNPs data. Contemporary effective population size (N_CUR_) was calculated and fixed in the models according to the equation NE = π/4μ^75^. Nucleotide diversity (π = 0.003 Hyp-C; π = 0.0008 Hyp-AP) was estimated using variant and invariant sites with DNAsp v12.03^76^, and the empirical mutation rate (μ) of 2.8 × 10^–9^ estimated in *Drosophila melanogaster* Meigen (Diptera: Drosophilidae)^77^. The folded SFS for a single population was built considering a single SNP per locus using a Python script by Isaac Overcast (https://github.com/isaacovercast/easySFS). Although the cactus pest was first reported in Puerto Rico in 2005^26^, we used a wide bound for T_BOT_ estimation (between 50 and 1000 generations ago), since cactus infestations produced by *Hypogeococcus* spp. have been reported since 1951 in the USA (see Introduction section for more details on historical records). The same wide bounds to T_BOT_ (50–1000 generations ago) were set for Hyp-AP whose introduction is also unknown, but its first detection in the region dates back to 1996^31^.

Models were run independently using 100 replicates for each model, 40 cycles of the Brent algorithm, and 100,000 simulations for the calculation of the composite likelihood. The best demographic model was identified using Akaike's information criterion (AIC). AIC values were rescaled in terms of AIC differences (Δi) according to the formula: Δi = AICi − AICmin to compare between models. The model with a ΔAIC value of 0 and the highest AIC weight (ωi) were considered as the best. Finally, a parametric bootstrapping approach was used to construct 95% confidence intervals of the estimated parameters running 100 bootstrap replicates using initialized values from the best model^37^.

### Assessment of host plant attack

The presence of Hyp-C mealybugs was detected in 12 out of 39 sites examined with Cactaceae. We searched for Hyp-AP in 28 sites with either Amaranthaceae and/or Portulacaceae and found mealybugs in 17 sites. In sites where mealybugs were present, the incidence of host plant infestation produced by Hyp-C or Hyp-AP were evaluated, along with a determination of whether the proportion of hosts attacked varied between species and among sites within each putative species. A generalized linear model (GLM) with quasi-binomial error distribution and logit link function was used to compare the proportion of hosts attacked between species. Statistical analysis was performed using the R version 4.1.1^78^. Host plant infestation by Hyp-C or Hyp-AP throughout the island was analyzed using the Neu method^79^ by comparing host plant availability with the actual proportion of plants attacked by Hyp-C or Hyp-AP, using a chi-square goodness of fit test. Assumptions of the statistical model were: (1) each attack on a host plant species found in the field was considered an independent observation; (2) availability of each host plant was the same for all mealybugs. Two null hypotheses tested were: (1) use of each host plant by Hyp-C/Hyp-AP is proportional to their respective availability, considering all host plants present in each sampling site simultaneously; and (2) Hyp-C/Hyp-AP use host plants in proportion to their availability, considering all of the host plants separately. When a difference in host use was detected (null hypothesis 1), simultaneous confidence intervals (CI) were calculated utilizing the following Bonferroni t-statistic to test the second hypothesis:

$$CI = \hat{p}_{j} \pm t_{ \propto /2k.n - 1} \sqrt {\hat{p}_{j} \left( {1 - \hat{p}_{j} } \right)/n}$$

where $\hat{p}_{j}$ is the proportion of plants used by Hyp-C/Hyp-AP and the $\propto /2k$ is used to ensure that *k* (number of plants analyzed) simultaneous CIs have an overall = 0.05. Thus, three scenarios of host use were established: (1) preference, when host use was higher than its availability (i.e., plant availability was lower than the lower limit of the CI); (2) proportional use, when host use was proportional to host availability (host plant availability was within the CI); and (3) avoidance, when host use was lower than host availability (i.e., plant availability was higher than the upper limit of the CI).

### Ecological niche modeling

Maps and correlative distribution models based on environmental variables were made to study the potential geographic distribution of both *Hypogeococcus* species in Puerto Rico. The conceptual framework we assumed for ecological niche models is described by Soberón & Peterson^80^ and Soberón & Nakamura^81^. In general, the distribution area of a species is the result of the combination of at least three fundamental factors; the biotically favorable areas for the species to occur, the environmentally favorable conditions for survival and reproduction of the species, and the accessible areas to the species in which it has been able to disperse. The distribution models were generated using a maximum entropy algorithm in MaxEnt version 3.4.1^82^ by selecting and optimizing model parameters in the R package “Kuenm”^83^. Projections to a future intermediate scenario were made for the time periods 2050 (average for 2041–2060) and 2070 (average for 2061–2080). In this scenario, greenhouse emissions in the Representative Concentration Pathway (RCP) 4.5 are expected to reach its maximum around 2040, and then to decline.

Occurrence records of presumptive *Hypogeococcus* species studied herein were obtained from field surveys conducted in 2016 and 2018 (Supplementary Table S1). The environmental variables used for ecological niche modeling were the bioclimatic variables from WorldClim v2.0^84^ and four variables from Envirem^85^. All variables were trimmed to Puerto Rico extent and then selected using a variance inflation factor (VIF) criterion (variables with VIF > 10 were removed). After selection, the variables used to construct the models were; Annual Potential Evapotranspiration (annPET), Thornthwaite Aridity Index (aridIndx), Mean Temperature of Wettest Quarter (bio08), and Precipitation of Wettest Month (bio13). Variables for future scenarios were the variables from CIMP5 of WorldClim. Details about occurrence records, environmental variables selection, and data processing can be found in supplementary information (Methods S1).

Candidate models were made in Kuenm using a combination of parameters consisting of 11 types of regularization multipliers (0.1, 0.3, 0.4, 0.5, 0.6, 0.7, 0.8, 0.9, 1, 2, 3) 29 combinations of response classes or model function (l = linear, q = quadratic, p = product, t = threshold, and h = hinge) and the four selected environmental variables. Model performance was evaluated on the basis of statistical significance (partial ROC), omission rates (OR) and Akaike's information criterion corrected for small sample sizes (AICc). Models with an OR > 0.05 were considered as low performing and therefore excluded; the remaining models were filtered according to the AICc. The final models were created using the best model parameter settings, performing 10 replications and employing a cloglog output, which was used to plot the final models. A niche identity or equivalency test^86^ were made to estimate the overlap between the two ecological niche models using the ENMTools package of the R environment^87^. In this test the overlap between models is quantified using the "Schoener's D"^88^ and "Hellinger's I"^89^ indices. These take values between 0 and 1, indicating the degree of overlap between the two models, where a value of 0 indicates the absence of overlap and a value of 1 the total overlap.

## Supplementary Information

Supplementary Information.

## Data Availability

The datasets generated during the current study, R code of genetic and ecological analyses as well as demographic models used in Fastsimcoal2 are available in Figshare^35^. Raw reads are available at NCBI as BioProject PRJNA593002. The reference draft genome is available at the NCBI under accession number JAAOIU000000000. *Hypogeococcus* occurrence records are available in supplementary information.
