# Supplementary material for: Genomic and ecological evidence shed light on the recent demographic history of two related invasive insects
Source: Sci Rep. 2022 Nov 16;12:19629. doi: 10.1038/s41598-022-21548-y (PMC9669014; doi:10.1038/s41598-022-21548-y)
Supplement: Supplementary file 1 — Supplementary Information. [file 41598_2022_21548_MOESM1_ESM.pdf]

Supplementary Information for:

Genomic and ecological evidence shed light on the recent demographic history of two related invasive insects

Daniel Poveda-Martínez, Nicolás A. Salinas, María Belén Aguirre, Andrés F. Sánchez Restrepo, Stephen Hight, Hilda Diaz-Soltero, Guillermo Logarzo, & Esteban Hasson.

Table of contents

Table S1. Population sampling of both invasive species: Hyp-C and Hyp-AP.

Table S2. Number of SNPs retained after each quality filtering step.

Table S3. Diversity estimates after normalization of sample size.

Table S4. Comparison between alternative demographic models.

Table S5. Host plant infestation produced by both invasive species: Hyp-C and Hyp-AP.

Table S6. Comparison between alternative ecological niche models.

Figure S1. PCA's after removal of SNPs with population-specific alleles.

Figure S2. Identity test for both invasive species ENMs: Hyp-C and Hyp-AP.

Methods S1. Methodological details about ecological niche models.

Table S1. Localities and host plants on which specimens of both *Hypogeococcus* presumptive species were sampled in Puerto Rico and adjacent islands, and used in population genomics and ecological surveys.

Species	Country	Island	Location site	Latitude	Longitude	Host plant species	Host plant family	Status	Date
Hyp-C	Puerto Rico	Caja de Muertos	Caja de Muertos	1.789.625	-6.652.170	*Stenocereus fimbriatus*	Cactaceae	Pest	2/19/2016
Hyp-C	Puerto Rico	Caja de Muertos	Caja de Muertos	1.789.625	-6.652.170	*Stenocereus fimbriatus*	Cactaceae	Pest	2/19/2016
Hyp-C	Puerto Rico	Caja de Muertos	Caja de Muertos	1.789.625	-6.652.170	*Stenocereus fimbriatus*	Cactaceae	Pest	2/19/2016
Hyp-C	Puerto Rico	Caja de Muertos	Caja de Muertos	1.789.625	-6.652.170	*Stenocereus fimbriatus*	Cactaceae	Pest	2/19/2016
Hyp-C	Puerto Rico	Caja de Muertos	Caja de Muertos	1.789.625	-6.652.170	*Stenocereus fimbriatus*	Cactaceae	Pest	2/19/2016
Hyp-C	Puerto Rico	Main Island	Cabo Rojo	1.797.880	-6.716.903	*Pilosocereus royenii*	Cactaceae	Pest	02/11/2016
Hyp-C	Puerto Rico	Main Island	Cabo Rojo	1.797.880	-6.716.903	*Pilosocereus royenii*	Cactaceae	Pest	02/11/2016
Hyp-C	Puerto Rico	Main Island	Cabo Rojo	1.797.880	-6.716.903	*Pilosocereus royenii*	Cactaceae	Pest	02/11/2016
Hyp-C	Puerto Rico	Main Island	Cabo Rojo	1.797.880	-6.716.903	*Pilosocereus royenii*	Cactaceae	Pest	02/11/2016
Hyp-C	Puerto Rico	Main Island	Cabo Rojo	1.797.880	-6.716.903	*Pilosocereus royenii*	Cactaceae	Pest	02/11/2016
Hyp-C	Puerto Rico	Main Island	Cabo Rojo	1.797.880	-6.716.903	*Pilosocereus royenii*	Cactaceae	Pest	02/11/2016
Hyp-C	Puerto Rico	Main Island	Cabo Rojo	1.798.111	-6.717.056	*Pilosocereus royenii*	Cactaceae	Pest	3/28/2018
Hyp-C	Puerto Rico	Main Island	Cabo Rojo	1.795.667	-6.719.972	*Pilosocereus royenii*	Cactaceae	Pest	3/29/2018
Hyp-C	Puerto Rico	Main Island	Cabo Rojo	1.794.138	-6.719.221	*Pilosocereus royenii*	Cactaceae	Pest	04/09/2018
Hyp-C	Puerto Rico	Main Island	Cabo Rojo	1.797.860	-6.717.117	*Pilosocereus royenii*	Cactaceae	Pest	04/09/2018
Hyp-C	Puerto Rico	Main Island	Cabo Rojo	1.797.860	-6.717.117	*Pilosocereus royenii*	Cactaceae	Pest	04/09/2018
Hyp-C	Puerto Rico	Main Island	Cabo Rojo	1.798.028	-6.717.056	*Pilosocereus royenii*	Cactaceae	Pest	3/28/2018
Hyp-C	Puerto Rico	Main Island	Cabo Rojo	1.797.880	-6.716.903	*Stenocereus fimbriatus*	Cactaceae	Pest	02/11/2016
Hyp-C	Puerto Rico	Main Island	Cabo Rojo	1.797.877	-6.716.907	*Stenocereus fimbriatus*	Cactaceae	Pest	04/09/2018
Hyp-C	Puerto Rico	Main Island	Cabo Rojo	1.797.877	-6.716.907	*Stenocereus fimbriatus*	Cactaceae	Pest	04/09/2018

Hyp-C	Puerto Rico	Main Island	Guánica	1.795.911	-6.686.148	*Melocactus intortus*	Cactaceae	Pest	2/14/2016
Hyp-C	Puerto Rico	Main Island	Guánica	1.795.027	-6.683.923	*Melocactus intortus*	Cactaceae	Pest	04/09/2018
Hyp-C	Puerto Rico	Main Island	Guánica	1.796.014	-6.686.125	*Melocactus intortus*	Cactaceae	Pest	04/09/2018
Hyp-C	Puerto Rico	Main Island	Guánica	1.796.014	-6.686.125	*Melocactus intortus*	Cactaceae	Pest	04/09/2018
Hyp-C	Puerto Rico	Main Island	Guánica	1.795.806	-6.686.500	*Pilosocereus royenii*	Cactaceae	Pest	3/27/2018
Hyp-C	Puerto Rico	Main Island	Guánica	1.795.911	-6.686.148	*Pilosocereus royenii*	Cactaceae	Pest	2/14/2016
Hyp-C	Puerto Rico	Main Island	Guánica	1.795.911	-6.686.148	*Pilosocereus royenii*	Cactaceae	Pest	2/14/2016
Hyp-C	Puerto Rico	Main Island	Guánica	1.795.911	-6.686.148	*Pilosocereus royenii*	Cactaceae	Pest	2/14/2016
Hyp-C	Puerto Rico	Main Island	Guánica	1.795.911	-6.686.148	*Pilosocereus royenii*	Cactaceae	Pest	2/14/2016
Hyp-C	Puerto Rico	Main Island	Guánica	1.795.911	-6.686.148	*Pilosocereus royenii*	Cactaceae	Pest	2/14/2016
Hyp-C	Puerto Rico	Main Island	Guánica	1.795.027	-6.683.923	*Pilosocereus royenii*	Cactaceae	Pest	04/09/2018
Hyp-C	Puerto Rico	Main Island	Guánica	1.795.027	-6.683.923	*Pilosocereus royenii*	Cactaceae	Pest	04/09/2018
Hyp-C	Puerto Rico	Main Island	Guánica	1.796.014	-6.686.125	*Pilosocereus royenii*	Cactaceae	Pest	04/09/2018
Hyp-C	Puerto Rico	Main Island	Punta Petrona	1.795.223	-6.638.332	*Hylocereus trigonus*	Cactaceae	Pest	2/13/2016
Hyp-C	Puerto Rico	Main Island	Punta Petrona	1.795.223	-6.638.332	*Hylocereus trigonus*	Cactaceae	Pest	2/13/2016
Hyp-C	Puerto Rico	Main Island	Punta Petrona	1.795.223	-6.638.332	*Hylocereus trigonus*	Cactaceae	Pest	2/13/2016
Hyp-C	Puerto Rico	Main Island	Punta Petrona	1.795.223	-6.638.332	*Hylocereus trigonus*	Cactaceae	Pest	2/13/2016
Hyp-C	Puerto Rico	Main Island	Punta Petrona	1.795.302	-6.638.361	*Hylocereus trigonus*	Cactaceae	Pest	04/08/2018
Hyp-C	Puerto Rico	Main Island	Punta Petrona	1.795.302	-6.638.361	*Hylocereus trigonus*	Cactaceae	Pest	04/08/2018
Hyp-C	Puerto Rico	Main Island	Punta Petrona	1.795.223	-6.638.332	*Pilosocereus royenii*	Cactaceae	Pest	02/10/2016
Hyp-C	Puerto Rico	Main Island	Punta Petrona	1.795.223	-6.638.332	*Pilosocereus royenii*	Cactaceae	Pest	02/10/2016
Hyp-C	Puerto Rico	Main Island	Punta Petrona	1.795.223	-6.638.332	*Pilosocereus royenii*	Cactaceae	Pest	04/08/2018
Hyp-C	Puerto Rico	Main Island	Punta Petrona	1.795.223	-6.638.332	*Pilosocereus royenii*	Cactaceae	Pest	04/08/2018
Hyp-C	Puerto Rico	Main Island	Punta Petrona	1.795.223	-6.638.332	*Pilosocereus royenii*	Cactaceae	Pest	04/08/2018

Hyp-AP	Puerto Rico	Culebra	Culebra town	1.830.128	-6.529.952	*Achyranthes aspera var. aspera*	Amaranthaceae	Invasive	2/18/2016
Hyp-AP	Puerto Rico	Culebra	Punta Soldado	1.828.195	-6.528.657	*Achyranthes aspera var. aspera*	Amaranthaceae	Invasive	10/04/2018
Hyp-AP	Puerto Rico	Culebrita	Culebrita Island	1.832.302	-6.522.784	*Alternanthera crucis*	Amaranthaceae	Invasive	2/18/2016
Hyp-AP	Puerto Rico	Culebrita	Culebrita Island	1.832.333	-6.522.775	*Alternanthera crucis*	Amaranthaceae	Invasive	04/11/2018
Hyp-AP	Puerto Rico	Culebrita	Culebrita Island	1.832.333	-6.522.775	*Alternanthera crucis*	Amaranthaceae	Invasive	04/11/2018
Hyp-AP	Puerto Rico	Culebrita	Culebrita Island	1.832.333	-6.522.775	*Alternanthera crucis*	Amaranthaceae	Invasive	04/11/2018
Hyp-AP	Puerto Rico	Culebrita	Culebrita Island	1.832.333	-6.522.775	*Alternanthera crucis*	Amaranthaceae	Invasive	04/11/2018
Hyp-AP	Puerto Rico	Culebrita	Culebrita Island	1.832.333	-6.522.775	*Alternanthera crucis*	Amaranthaceae	Invasive	04/11/2018
Hyp-AP	Puerto Rico	Culebrita	Culebrita Island	1.832.333	-6.522.775	*Alternanthera crucis*	Amaranthaceae	Invasive	04/11/2018
Hyp-AP	Puerto Rico	Culebrita	Culebrita Island	1.832.302	-6.522.784	*Portulaca cf pilosa*	Portulacaceae	Invasive	2/18/2016
Hyp-AP	Puerto Rico	Culebrita	Culebrita Island	1.832.302	-6.522.784	*Portulaca cf pilosa*	Portulacaceae	Invasive	2/18/2016
Hyp-AP	Puerto Rico	Culebrita	Culebrita Island	1.832.302	-6.522.784	*Portulaca cf pilosa*	Portulacaceae	Invasive	2/18/2016
Hyp-AP	Puerto Rico	Culebrita	Culebrita Island	1.832.302	-6.522.784	*Portulaca cf pilosa*	Portulacaceae	Invasive	2/18/2016
Hyp-AP	Puerto Rico	Culebrita	Culebrita Island	1.832.333	-6.522.775	*Portulaca cf pilosa*	Portulacaceae	Invasive	04/11/2018
Hyp-AP	Puerto Rico	Main Island	Punta Pozuelo	1.793.703	-6.618.419	*Gomphrena serrata*	Amaranthaceae	Invasive	04/07/2018
Hyp-AP	Puerto Rico	Main Island	Punta Pozuelo	1.793.703	-6.618.419	*Gomphrena serrata*	Amaranthaceae	Invasive	04/07/2018
Hyp-AP	Puerto Rico	Main Island	Sector Hucar	1.801.182	-6.625.125	*Achyranthes aspera var. aspera*	Amaranthaceae	Invasive	04/07/2018
Hyp-AP	Puerto Rico	Main Island	Sector Hucar	1.801.245	-6.625.148	*Portulaca teretifolia*	Portulacaceae	Invasive	02/10/2016
Hyp-AP	Puerto Rico	Main Island	Sector Hucar	1.801.245	-6.625.148	*Portulaca teretifolia*	Portulacaceae	Invasive	02/10/2016
Hyp-AP	Puerto Rico	Main Island	Sector Hucar	1.801.245	-6.625.148	*Portulaca teretifolia*	Portulacaceae	Invasive	02/10/2016
Hyp-AP	Puerto Rico	Main Island	Sector Hucar	1.801.182	-6.625.125	*Portulaca teretifolia*	Portulacaceae	Invasive	04/07/2018
Hyp-AP	Puerto Rico	Main Island	Sector Hucar	1.801.182	-6.625.125	*Portulaca teretifolia*	Portulacaceae	Invasive	04/07/2018
Hyp-AP	Puerto Rico	Mona Island	Lighthouse	1.808.492	-6.785.117	*Portulaca caulerpoides*	Portulacaceae	Invasive	02/12/2016
Hyp-AP	Puerto Rico	Mona Island	Lighthouse	1.806.303	-6.787.195	*Amaranthaceae*	Amaranthaceae	Invasive	02/12/2016

Hyp-AP	Puerto Rico	Mona Island	Lighthouse	1.807.346	-6.786.453	*Portulaca caulerpoides*	Portulacaceae	Invasive	02/12/2016
Hyp-AP	Puerto Rico	Mona Island	Lighthouse	1.807.346	-6.786.453	*Portulaca rubricaulis*	Portulacaceae	Invasive	02/12/2016
Hyp-AP	Puerto Rico	Vieques	Biological Station	1.812.195	-6.541.605	*Achyranthes aspera var. aspera*	Amaranthaceae	Invasive	2/17/2016
Hyp-AP	Puerto Rico	Vieques	Biological Station	1.812.195	-6.541.605	*Achyranthes aspera var. aspera*	Amaranthaceae	Invasive	2/17/2016
Hyp-AP	Puerto Rico	Vieques	Biological Station	1.812.195	-6.541.605	*Achyranthes aspera var. aspera*	Amaranthaceae	Invasive	2/17/2016
Hyp-AP	Puerto Rico	Vieques	Punta Conejo	1.810.976	-6.537.744	*Portulaca rubricaulis*	Portulacaceae	Invasive	2/17/2016
Hyp-AP	Puerto Rico	Vieques	Heliport Monte Pirata	1.809.366	-6.555.117	*Achyranthes aspera var. aspera*	Amaranthaceae	Invasive	4/13/2018
Hyp-AP	Puerto Rico	Vieques	Heliport Monte Pirata	1.809.366	-6.555.117	*Portulaca teretifolia*	Portulacaceae	Invasive	4/13/2018
Hyp-AP	Puerto Rico	Vieques	Monte Pirata	1.809.362	-6.555.121	*Achyranthes aspera var. aspera*	Amaranthaceae	Invasive	2/17/2016
Hyp-AP	Puerto Rico	Vieques	Monte Pirata	1.809.362	-6.555.121	*Achyranthes aspera var. aspera*	Amaranthaceae	Invasive	2/17/2016
Hyp-AP	Puerto Rico	Vieques	Monte Pirata	1.809.362	-6.555.121	*Achyranthes aspera var. aspera*	Amaranthaceae	Invasive	2/17/2016
Hyp-AP	Puerto Rico	Vieques	Monte Pirata	1.809.362	-6.555.121	*Achyranthes aspera var. aspera*	Amaranthaceae	Invasive	2/17/2016
Hyp-AP	Puerto Rico	Vieques	Monte Pirata	1.809.362	-6.555.121	*Portulaca oleracea*	Portulacaceae	Invasive	2/17/2016
Hyp-AP	Puerto Rico	Vieques	Monte Pirata	1.809.653	-6.552.499	*Achyranthes aspera var. aspera*	Amaranthaceae	Invasive	4/13/2018
Hyp-AP	Puerto Rico	Vieques	Monte Pirata	1.809.653	-6.552.499	*Achyranthes aspera var. aspera*	Amaranthaceae	Invasive	4/13/2018
Hyp-AP	Puerto Rico	Vieques	Monte Pirata	1.809.653	-6.552.499	*Portulaca oleracea*	Portulacaceae	Invasive	4/13/2018
Hyp-AP	Puerto Rico	Vieques	Monte Pirata	1.809.653	-6.552.499	*Portulaca teretifolia*	Portulacaceae	Invasive	4/13/2018
Hyp-AP	Puerto Rico	Vieques	Monte Pirata	1.811.389	-6.554.765	*Portulaca teretifolia*	Portulacaceae	Invasive	2/17/2016
Hyp-AP	Puerto Rico	Vieques	Playa Escondida	1.811.533	-6.537.719	*Portulaca teretifolia*	Portulacaceae	Invasive	4/13/2018
Hyp-AP	Puerto Rico	Vieques	Punta Conejo	1.810.796	-6.537.565	*Achyranthes aspera var. aspera*	Amaranthaceae	Invasive	04/12/2018
Hyp-AP	Puerto Rico	Vieques	Punta Conejo	1.810.796	-6.537.565	*Achyranthes aspera var. aspera*	Amaranthaceae	Invasive	04/12/2018

Table S2. Number of SNPs retained after quality filtering steps for both *Hypogeococcus* species invading Puerto Rico (Hyp-C and Hyp-AP).

Filtering steps	Retained SNPs	
	Hyp-C	Hyp-AP
Initial count after variant calling	39,266	61,270
Biallelic SNPs only	37,663	59,188
Min-max mean depth (6X - 100X)	12,772	19,713
Genotyped successfully in 90% of individuals	5,804	11,061
MAF < 0,03	1,802	1,807
Monomorphic SNPs after removal of samples	1,524	1,783
SNPs under linkage disequilibrium	1,524	1,292
Outliers SNPs	1,524	1,284
Total SNPs used in analyses	1,524	1,284

Table S3. Genetic diversity statistics normalized to the number of individuals for interpopulation comparison in both species (Hyp-C and Hyp-AP). N: sample size; H_E: expected heterozygosity; H_O: observed heterozygosity; A_R: allelic richness.

Species	N	HE	HO	AR
**Hyp-C**				
Cabo Rojo	5	0.182	0.216	1.209
Caja de Muertos	5	0.122	0.154	1.137
Guánica	5	0.204	0.255	1.231
Punta Petrona	5	0.154	0.169	1.174
**Hyp-AP**				
Hucar	4	0.120	0.143	1.141
Vieques (W)	4	0.114	0.141	1.133
Vieques (E)	4	0.110	0.132	1.128
Culebrita	4	0.138	0.170	1.650

Table S4. Comparisons between alternative demographic models for each *Hypogeococcus* species invading Puerto Rico (Hyp-C and Hyp-AP) using Fastsimcoal2. K: number of parameters estimated; log10L: maximum likelihood of the model; AIC: Akaike's information criterion; ΔAIC: difference in AIC scores of each model and the strongest model; ωi; AIC weight. The best models are indicated in bold.

Specie	K	log10L	AIC	ΔAIC	*ωi*
**Hsp-C**					
Model A	1	-6868.95	31636.69	6859.98	0
Model B	2	-5378.89	**24776.71**	0	0.74
Model C	3	-5378.91	**24778.78**	2.06	0.26
Model D	4	-5387.91	24822.26	45.54	0
**Hsp-AP**					
Model A	1	-10982.99	50582.55	18225.36	0
Model B	2	-7024.97	**32357.2**	0	0.86
Model C	3	-7025.75	**32360.77**	3.57	0.14
Model D	4	-7142.04	32900.31	543.11	0

Table S5. Patterns of host plant infestation produced by both *Hypogeococcus* species (Hyp-C and Hyp-AP) in each sampled locality in Puerto Rican islands.

Species	Site	Host plant species	Proportion of plants available	Confidence interval of plant utilization	Host use
**Hyp-C**	Guánica (X^2 = 13.625, *df* = 1, *P* < 0.01)	*Melocactus intortus*	0.669	0.332–0.618	Avoided
		Pilosocereus royenii	0.331	0.382–0.668	Preferred
	Cabo Rojo (X^2 = 0.297, *df* = 1, *P* > 0.05)	*Pilosocereus royenii*	0.886	-	Random
		Stenocereus fimbriatus	0.114	-	Random
	Punta Petrona (X^2 = 10.464, *df* = 1, *P* < 0.01)	*Pilosocereus royenii*	0.75	0.795–0.939	Preferred
		Hylocereus trigonus	0.25	0.061–0.205	Avoided
	Caja de Muertos	*Stenocereus fimbriatus*	-	-	-
**Hyp-AP**	Culebrita (X^2 = 2.062, *df* = 2, *P* > 0.05)	*Portulaca cf pilosa*	0.631	-	Random
		Portulaca oleracea	0.023	-	Random
		Alternanthera crucis	0.346	-	Random
	Hucar (X^2 = 0.488, *df* = 1, *P* > 0.05)	*Portulaca teretifolia*	0.758	-	Random
		Achyranthes aspera var. aspera	0.242	-	Random
	East Vieques (X^2 = 72.145, *df* = 3, *P* < 0.01)	*Achyranthes aspera var. aspera*	0.596	0.127–0.421	Avoided
		Portulaca oleracea	0.084	0.000–0.000	Avoided
		Portulaca rubricaulis	0.262	0.328–0.658	Preferred
		Portulaca teretifolia	0.058	0.094–0.372	Preferred
	West Vieques (X^2 = 7.371, *df* = 3, *P* > 0.05)	*Achyranthes aspera var. aspera*	0.45	-	Random
		Portulaca oleracea	0.072	-	Random
		Portulaca rubricaulis	0.039	-	Random
		Portulaca teretifolia	0.44	-	Random
	Mona (X^2 = 42.267 , *df* = 2, *P* < 0.01)	*Portulaca caulerpoides*	0.649	0.051–0.393	Avoided
		Portulaca rubricaulis	0.325	0.607–0.949	Preferred
		Portulaca oleracea	0.026	0.000–0.000	Avoided
	Punta Pozuelo (X^2 =0.857, *df* = 1, *P* > 0.05)	*Gomphrena serrata*	0.538	-	Random
		Portulaca sp.	0.462	-	Random
	Culebra	*Achyranthes aspera var. aspera*	-	-	-

Table S6. Performance statistics of the best models selected based on the number of significant models that met the 5% omission criterion and the statistically significant models that met the AICc criteria for both *Hypogeococcus* species (Hyp-C and Hyp-AP).

Species	Model	Mean AUC ratio	pval ROC	Omission rate at 5%	AICc	ΔAICc	AICc weight	Number of parameters
Hyp-C	M_0.6_F_pt	1.944	0	0.333	199.947	0.000	1	7
Hyp-C	M_0.6_F_lpt	1.943	0	0.333	199.965	0.018	1	7
Hyp-C	M_0.7_F_pt	1.935	0	0.333	201.946	1.999	1	6
Hyp-AP	M_0.1_F_lq	1.942	0	0.333	239.570	0.000	1	6
Hyp-AP	M_1_F_lt	1.839	0	0.333	240.522	0.952	1	4
Hyp-AP	M_0.8_F_lt	1.850	0	0.333	241.301	1.730	1	5

Figure S1. Results of PCAs after removal of SNPs with population-specific alleles for (A) the cactus pest: Hyp-C and (B) the Amaranthaceae and Portulacaceae feeding mealybugs: Hyp-AP.

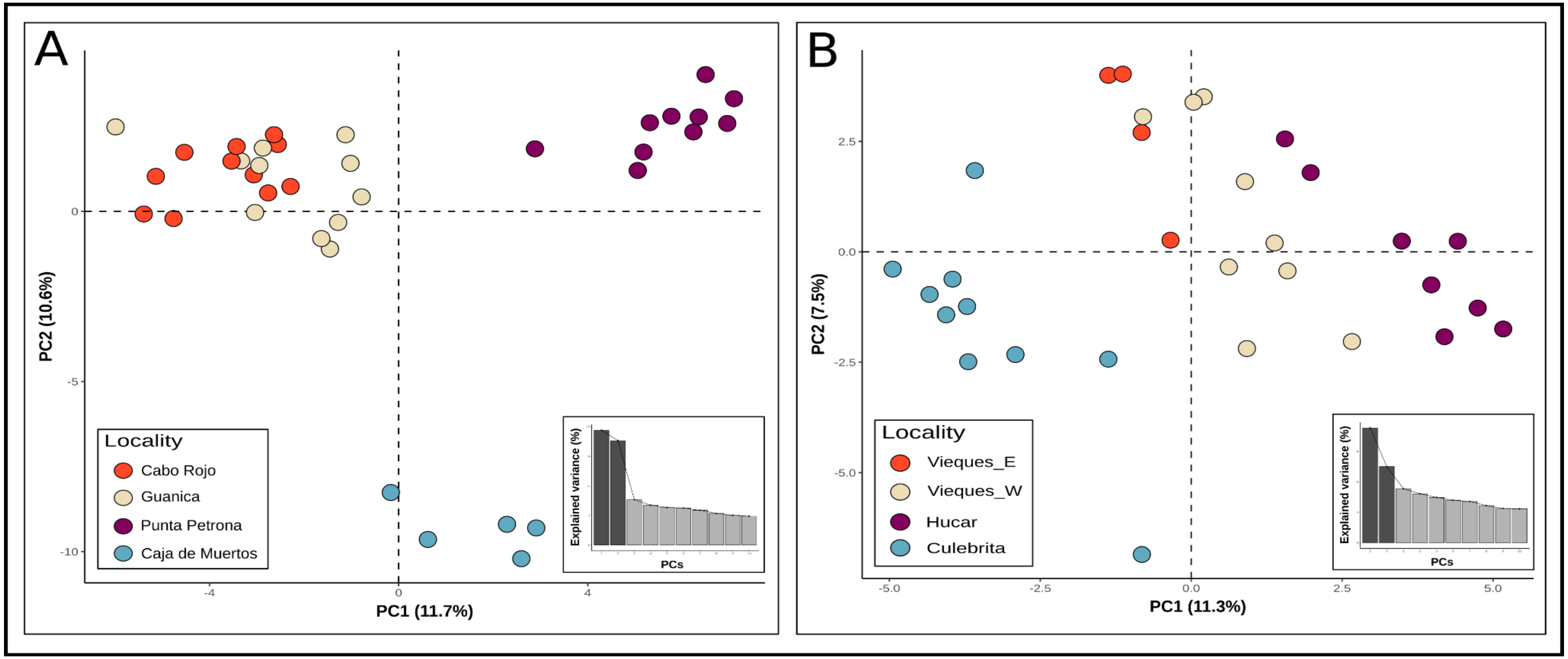

Figure S2. Identity test (or equivalency test) between ENMs using the "Schoener's D" and "Hellinger's I" indices. Plots represent the probability distribution for niche model overlap calculated between species under the null hypothesis that the two Hypogeococcus species (Hyp-C and Hyp-AP) occurrences in the environment are effectively a random draw from the same underlying distribution.

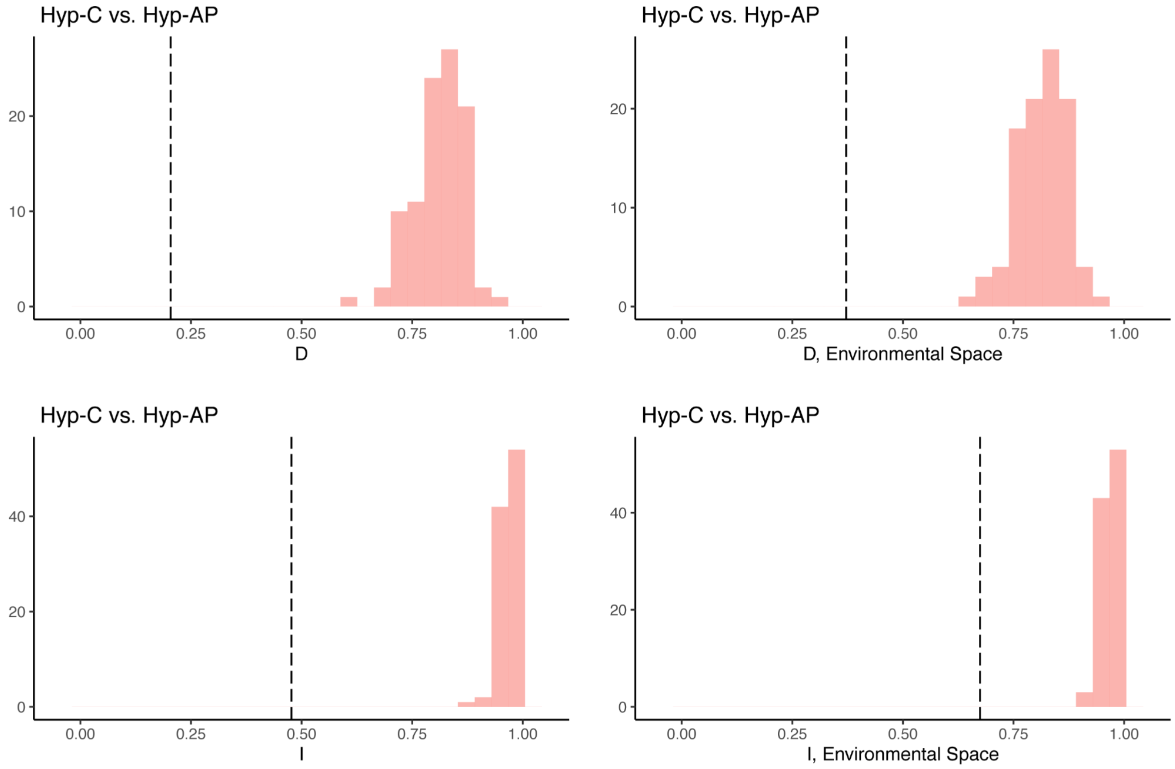

Method S1. Methodological details about ecological niche models

All occurrence records used to produce the models are presented in Table S1. For both species duplicates were removed. To calibrate the models, occurrences were split in 20% for testing and 80% to train the models. The environmental variables used for ecological niche modeling were the bioclimatic variables retrieved from WorldClim v2.0 (Fick & Hijmans 2017) and four variables from Envirem (Title & Bemmels 2018). From these sets of variables four of the least correlated variables were selected. To this end, a variance inflation factor (VIF) was calculated for each variable using the *vif* function of the R package "usdm" (Naimi & Araújo 2016). The VIF provides a measure of multicollinearity among independent variables in a multiple regression model. The VIF can be used to identify and discard collinear predictors by sequentially removing the covariate with the highest VIF, using a threshold of 10, and by repeating this process until all VIFs are smaller than the threshold (Zuur & Ieno 2016). Niche overlap between Hyp-C and Hyp-AP was estimated using "Schoener's D" (Schoener, 1968) and "Hellinger's I" (van der Vaart, 1998), a value of 0 indicates the absence of overlap and 1 total overlap. In this test the probability distribution for niche model overlap was calculated between species under the null hypothesis that the two species occurrences in the environment are effectively a random draw from the same underlying distribution (Warren et al. 2008).
